# Patient-reported side effects, concerns and adherence to corticosteroid treatment for asthma, and comparison with physician estimates of side-effect prevalence: a UK-wide, cross-sectional study

**DOI:** 10.1038/npjpcrm.2015.26

**Published:** 2015-07-09

**Authors:** Vanessa Cooper, Leanne Metcalf, Jenny Versnel, Jane Upton, Samantha Walker, Rob Horne

**Affiliations:** 1 Centre for Behavioural Medicine, UCL School of Pharmacy, London, UK; 2 Asthma UK, London, UK; 3 Education for Health, London, UK

## Abstract

**Background:**

Non-adherence to corticosteroid treatment has been shown to reduce treatment efficacy, thus compromising asthma control.

**Aims:**

To examine the experiences of treatment side effects, treatment concerns and adherence to inhaled (ICS) and oral corticosteroids (OCS) among people with asthma and to identify the degree of concordance between clinician estimates of side effects and the prevalence reported by patients.

**Methods:**

Asthma UK members were sent validated questionnaires assessing treatment concerns, experiences of side effects and adherence. Questionnaires measuring clinicians’ estimates of the prevalence of corticosteroid side effects were completed online.

**Results:**

Completed questionnaires were returned by 1,524 people taking ICS, 233 taking OCS and 244 clinicians (67% of clinicians were primary care nurses). Among people with asthma, 64% of those taking ICS and 88% of those taking OCS reported ⩾1 side effect. People reporting high adherence to ICS (*t*=−3.09, *P*<0.005) and those reporting low adherence to OCS (*t*=1.86, *P*<0.05; one-tailed test) reported more side effects. There was a disparity between clinicians’ estimates of the frequency of side effects and the frequency reported by people with asthma: e.g., although 46% of people taking ICS reported sore throat, clinicians estimated that this figure would be 10%. Patients who reported side effects had stronger concerns about both ICS (*r*=0.46, *P*<0.0001) and OCS (*r*=0.50, *P*<0.0001). Concerns about corticosteroids were associated with low adherence to ICS (*t*=6.90, *P*<0.0001) and OCS (*t*=1.71; *P*<0.05; one-tailed test).

**Conclusions:**

An unexpectedly large proportion of people with asthma experienced side effects and had strong concerns about their treatment, which compromised adherence. These findings have implications for the design of interventions to optimise asthma control through improved adherence.

## Introduction

Inhaled corticosteroids (ICS) are the anti-inflammatory drug of choice for asthma.^1,2^ In cases in which asthma control is not achieved with ICS, oral corticosteroids (OCS) are added.^2^ These medicines are remarkably effective when taken as prescribed.^3^ However, only a minority of adults with asthma achieve good asthma control.^4–7^

Non-adherence to corticosteroid treatment reduces efficacy, compromising asthma control.^8,9^ Non-adherence is often an intentional decision on the part of the patient, stemming from concerns about corticosteroids, doubts about the need for preventative treatment, and experiences of side effects.^10–16^ The Common Sense Model^17,18^ proposes that non-adherence stems from a lack of coherence between individuals’ beliefs about their illness, their experience of symptoms and the doctor’s instructions. Adherence is also influenced by patients’ ‘common sense’ evaluations of treatment, particularly how they judge their personal need for treatment relative to their concerns about potential adverse effects (the Necessity Concerns Framework).^19,20^ Furthermore, treatment concerns are influenced by the individual’s interpretation of symptom experiences. Previous research has shown that treatment concerns are associated with non-adherence to treatment for a range of conditions including asthma;^10,21–25^ however, no previous studies have explored relationships between experiencing side effects and concerns about corticosteroids.

Side effects from corticosteroid medicines may go unreported. Studies in other clinical areas have identified disconnects between patient and clinician beliefs about treatment.^26^ We also examined clinicians estimates of the frequency of corticosteroids side effects they would expect patients to experience, to identify disconnects between physicians estimates and patient reports.

The primary aim of this study was to examine the frequency of patient-reported side effects of ICS and OCS among a large sample of people with asthma. Secondary aims were to profile patients’ concerns about ICS and OCS and to examine the impact of concerns and side-effect experiences on reported adherence. We also examined the estimated frequency of side effects among asthma-treating clinicians and identified disconnects between clinician estimates and patient reports.

## Materials and methods

### Design and sample

This was a UK-wide, cross-sectional, questionnaire-based survey of symptom experiences, treatment concerns and adherence, including people with asthma and asthma-treating clinicians.

### Procedure

Questionnaires were mailed to Asthma UK members and made available on the Asthma UK website. The online survey was promoted to people with asthma in communications from Asthma UK. Asthma-treating clinicians (doctors, nurses and pharmacists) were notified of the study through communications from Asthma UK and Education for Health (a UK provider of respiratory education and training courses for health professionals). An advert and questionnaire were placed on the Education for Health website.

### Measures

Side effects, treatment concerns and adherence were measured by self-reported questionnaire.

#### Side effects

People with asthma: A list of commonly reported side effects was constructed on the basis of literature review and interviews with people with asthma. Participants were asked to indicate which of a list of symptoms (sore mouth or throat, oral thrush, abnormal weight gain, bruising, behaviour changes and dental problems) they had experienced, which they believed could be a side effect of using corticosteroids. Separate lists were given for ICS and OCS. Each side effect was scored ‘yes’ (1), ‘no’(0) or ‘Don’t know’ (0). Possible scores ranged from 0 to 6.

Clinicians: Clinicians were asked to estimate the percentage of their patients prescribed corticosteroids to treat asthma within the past 3 years who had experienced each side effect. Separate questionnaires were completed for ICS and OCS.

#### Concerns about corticosteroids

Concerns were measured using the Beliefs about Medicines Questionnaire,^27^ which has been validated for use in asthma.^10,25,28^ The Beliefs about Medicines Questionnaire-Concerns scale (13 items) encompassed concerns about adverse effects of corticosteroid treatments, including side effects, disruptive effects of treatment regimen on daily life, potential long-term effects and dependency. Participants rated their level of agreement with each of a series of statements on a scale ranging from strongly agree (scored 5) to strongly disagree (scored 1). A mean scale score was computed (range 1–5). The prevalence of each concern was calculated by dichotomising: responses ‘agree’ or ‘strongly agree’=1 and all other responses=0.

#### Adherence

The Medication Adherence Report Scale (MARS©R Horne) was used to reported adherence.^29,30^ For ICS, a 4-item scale was used. Participants were asked to indicate how often they forgot to take their medicines, stopped taking them for a while, decided to miss out a dose or took less than instructed. Possible scores ranged from 4 to 20, with higher scores indicating greater adherence. For OCS, an additional question asked how often people completed the treatment course. Possible scores ranged from 5 to 25. Participants were divided into high and low adherence groups on the basis of the distribution of scores.^28,31^ Two-thirds of the sample with the highest scores were considered to have high adherence (score ⩾18 for ICS; ⩾23 for OCS), and one-third with the lowest scores was considered to have low adherence.

### Statistical analysis

Analyses were conducted using PASW Statistics 18. Associations between symptoms and adherence and between concerns and adherence were explored using independent samples *t*-tests. Pearson’s correlations were used to explore associations between side effects and concerns. Differences in the estimates of side effects between doctors, nurses and pharmacists were explored using the Kruskal–Wallis test.

## Results

### People with asthma

Questionnaires were sent to Asthma UK’s membership of approximately 8,000 people. A total of 2,659 questionnaires were returned by people with asthma (initial response rate 33.2%): 1971 (74%) by post and 688 (26%) online. Thirty-five questionnaires were excluded because the participant was <16 years of age. Of those currently prescribed ICS (*n*=2,213), 1524 (68.9%) had complete data and were included in the analyses. Of those currently prescribed OCS (*n*=314), 233 (74.2%) had complete data and were included in the analysis. Table 1 shows summary statistics for the participants included in the final ICS and OCS samples.

### Clinicians

There were 700 visitors to the Survey Monkey questionnaire. Of those, 166 (23.7%) indicated that they had read the study information; 534 initiated the questionnaire; and 244 clinicians completed the questionnaires and were included in the analysis (Response rate 34.9%; Table 2).

### Adherence

The mean MARS score for ICS was 17.4 (s.d.=3.2); 555 (36%) participants scored <18 and formed the ‘low adherence’ group, whereas 969 (64%) participants scored ⩾18 and were considered ‘highly adherent’. There was a significant positive relationship between age and adherence (*r*=0.287, *P*<0.0001), but no association between adherence and gender (*P*=0.90).

The mean MARS score for OCS was 23 (s.d.=2.6); 73 (31%) participants scored <23 (low adherence), whereas 160 (69%) participants scored ⩾23 (high adherence). Neither age, nor gender had a significant impact on adherence (both *P*>0.1).

### Side effects

**Figure 1:**
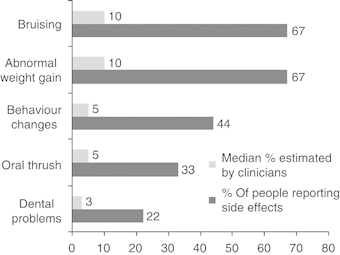
Disconnects between patient reports and clinician estimates of side effects from ICS. ICS, inhaled corticosteroid.

**Figure 2:**
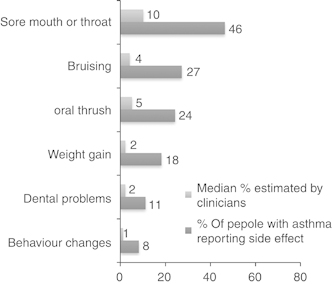
Disconnects between patient reports and clinician estimates of side effects from OCS. OCS, oral corticosteroid.

Sixty-four percent (*n*=971) of the sample reported ⩾1 side effect attributed to ICS (mean=1.3, s.d.=1.4), whereas 88% (*n*=205) reported ⩾1 side effect of OCS (mean=2.7, s.d.=1.7). The prevalence of each side effect attributed to ICS and OCS is shown in Figures 1 and 2, respectively. Older age (*r*=−0.139, *P*<0.000, *n*=1,524) and male gender (*t*=9.69, *P*<0.0001) were associated with fewer side effects of ICS (women: mean=1.6; s.d.=1.5; men: mean=0.8; s.d.=1.1). Older age (*r*=−0.215, *P*<0.01) and male gender (*t*=2.46, *P*<0.05) were also associated with fewer side effects of OCS (women: mean=2.9; s.d.=1.6; men: mean=2.2; s.d.=1.6).

### Clinician estimates of side effects

Comparisons between clinician estimates and patient reports of side effects from ICS and OCS are shown in Figures 1 and 2. There were no significant differences in the estimates between doctors, nurses and pharmacists (all *P*>0.1).

### Associations between side effects and adherence

People who were highly adherent reported a slightly greater number of ICS side effects than those with lower adherence (high adherence: mean=1.4, s.d.=1.5; low adherence: mean=1.2, s.d.=1.4; *t*=−3.09, *P*=0.002). Conversely, people who reported low adherence to OCS reported a greater number of side effects (low adherence: mean=3.0 symptoms, s.d.=1.7; high adherence: mean=2.6 symptoms, s.d.=1.7; *t*=1.86, *P*<0.05; one-tailed test).

### Concerns about corticosteroid medicines

**Figure 3:**
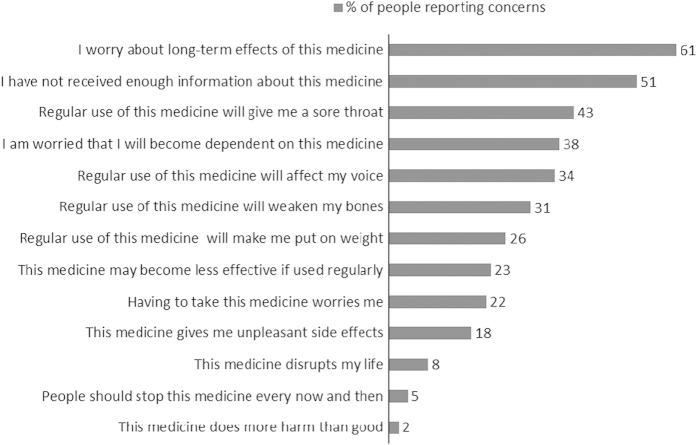
Percentage of people with asthma reporting specific concerns about ICS. ICS, inhaled corticosteroid. Questionnaire statements © R Horne.

**Figure 4:**
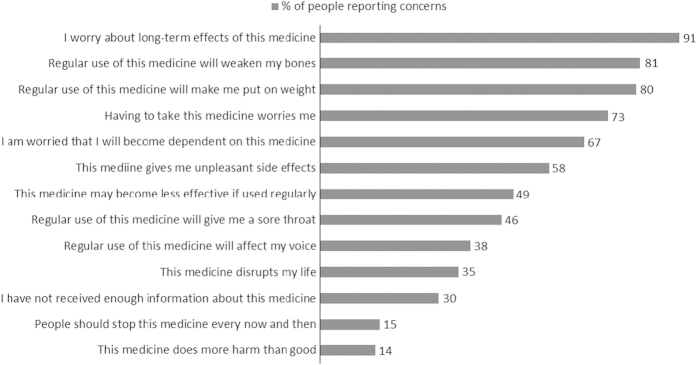
Percentage of people reporting concerns about OCS. OCS, oral corticosteroid. Questionnaire statements © R Horne.

Figures 3 and 4 show the percentage of participants reporting specific concerns about ICS and OCS.

There was a wide variation in concerns about ICS. Scores ranged from 1.0 to 4.8 (scale range=1–5; mean=2.5; s.d.=0.7). Older participants reported fewer concerns about ICS (*r*=−0.150; *P*<0.001), whereas women had stronger concerns about ICS than men (scale range=1–5; mean=3.4; s.d.=0.6, respectively (*t*=7.08, *P*<0.0001)). With regard to OCS, concerns scores ranged from 1.3 to 5.0 (mean=3.4; s.d.=0.6). Older participants reported fewer concerns about OCS (*r*=−0.182; *P*<0.01), whereas women had stronger concerns about OCS than men (mean=3.4 (s.d.=0.6) versus mean=3.1 (s.d.=0.6), respectively; *t*=2.89, *P*<0.005). Stronger concerns about ICS and OCS were associated with a greater number of side effects ((*r*=0.46, *P*<0.0001) and (*r*=0.50, *P*<0.0001), respectively).

### Associations between concerns about corticosteroids and adherence

#### ICS

People with stronger concerns about ICS and OCS were more likely to report low adherence (ICS—low adherence group: mean concerns score=2.7; s.d.=0.7; high adherence group: mean=2.4; s.d.=0.7 (*t*=6.90; df=1,522; *P*<0.0001); OCS—low adherence group: mean=3.5; s.d.=0.6; high adherence group: mean=3.3; s.d.=0.7 (*t*=1.71; df=231; *P*<0.05); one-tailed test).

## Discussion

### Main findings

We identified a high prevalence of reported side effects from both ICS and OCS in this large sample of people with asthma. There was a clear disconnect between clinician estimates of the prevalence of side effects and the actual prevalence reported by people with asthma. Consistent with our hypothesis, experiencing a greater number of side effects was associated with non-adherence to OCS. Conversely, those reporting a greater number of side effects were more likely to report high adherence to ICS. Reasons for this are unclear, but the finding may reflect a dose–response relationship between adherence and side effects of ICS. Consistent with previous studies, concerns about both OCS and ICS were associated with non-adherence.^10,25^ These findings indicate that, at least for patients taking ICS, their concerns about side effects, rather than the actual experience of side effects, may lead to non-adherence.^32^

In common with other studies,^33,34^ older participants were more adherent to ICS; however, the relationship between age and adherence has not been consistent across studies.^35^ Furthermore, male participants and those who were older reported significantly fewer side effects and had fewer concerns about their treatment. Although these results suggest that interventions to address concerns and to improve adherence may be of particular benefit to those who are younger, further research is required to confirm these findings.

### Strengths and limitations of this study

This study included a large sample of people with asthma and used validated questionnaires to measure perceptions of treatment and adherence. We were able to recruit a group of clinicians who estimated the prevalence of identical side effects to examine possible disconnects between the experiences of people taking corticosteroids and the perceptions of clinicians. The cross-sectional design meant that we were unable to examine changes in side-effect experiences over time or to infer the direction of relationships between side effects, concerns about corticosteroids and adherence. Participants recruited through Asthma UK may not have been representative of the UK population of people with asthma.

The majority of clinicians were nurses working in primary care, consistent with the model of nurse-led asthma clinics. However, relatively few doctors were included, limiting the extent to which these results can be applied to the doctor–patient setting. Because patient and clinician samples were unrelated, it was not possible to draw conclusions about the discrepancies in findings between the two groups.

Generalisability of the findings is also limited by low response rates and missing data. It is not possible to determine an exact response rate, as questionnaires were sent to all Asthma UK members, and we were unable to determine what proportion of those who did not return questionnaires were eligible for the study. Our final sample of people taking ICS was biased in terms of younger age and female gender. Given that those with missing data had stronger concerns about ICS than those included in the analysis, we hypothesise that the true prevalence of concerns about ICS is underestimated in our sample.

### Interpretation of findings in relation to previously published work

The prevalence of side effects in this study was higher than expected. Sore mouth and oral thrush are well-documented side effects of ICS.^36–38^ Skin thinning and bruising have also been previously associated with ICS.^39^ Other reported side effects to ICS, such as abnormal weight gain and behaviour changes, reported by a significant minority of people in this study are more commonly associated with OCS, but are rare in relation to ICS use.^40,41^ High doses of ICS over the long term might increase the risk of side effects associated with systemic use;^42^ however, we did not collect information on the type or dosage of ICS. The finding that people with higher rates of adherence to ICS reported more side effects is consistent with a dose–response relationship.

Although it is plausible that the high prevalence of side effects reported in this study was directly attributable to the pharmacological effects of corticosteroid medications, there may be other explanations. One alternative explanation is a phenomenon known as the ‘nocebo effect’, in which experiences of side effects stem from negative expectations of treatment, perhaps owing to negative past experiences of medicines or information about possible adverse reactions.^43^ The association between treatment concerns and experience of side effects lends support to this theory, and it is consistent with the findings of studies in other clinical areas.^14,15^ In this study, however, the direction of causality between concerns about corticosteroids and side effects could not be established.

In contrast to side-effect reports by people with asthma, the sample of clinicians felt that side effects from corticosteroids would be relatively infrequent among their patients, raising the question of whether people with asthma and clinicians view side effects in the same way. Discrepancies between clinicians’ and patients’ beliefs about medications have previously been reported.^26^ It may be that, although a large number of patients with asthma experience side effects of corticosteroids, few communicate them to clinicians.

### Implications for future research, policy and practice

Our findings are immediately relevant to asthma-treating clinicians and the design of interventions to promote adherence to corticosteroids. Eliciting and addressing patients’ concerns about their oral and inhaled corticosteroids may be an economical and clinically relevant way to facilitate adherence to prescriptions for OCS and ICS and thereby improve asthma control. This approach has previously been piloted in telephone-based medicines support intervention in which a pharmacist telephoned patients to elicit and address perceptual barriers and practical problems within 10 days of receiving new medicine. Patients receiving the intervention had fewer concerns, fewer medication problems and higher reported adherence than standard care controls.^44^ Randomised controlled trials to explore the efficacy of this approach in long-term illnesses are ongoing.

Clinicians should encourage patients to report new symptoms that they attribute to corticosteroids so that the likely cause, duration and possible treatment can be discussed. Common local side effects of ICS may be treated or prevented. For example, sore throat can be soothed by drinking fluids, gargling with warm salty water, lozenges or anaesthetic throat sprays,^45^ and oral thrush could be treated with mouthwashes or prevented by the use of a spacer device and mouth rinses with water.^36,37^ In cases in which it is not possible to prevent or treat side effects, clinicians could provide patients with accurate information about the risk of side effects, explore the likely causes of symptoms, and provide information on what to do if the patient experiences them, while emphasising the need for continued adherence. Given that there are differences between different inhalers with regard to their side-effect profiles, switching to a different type of ICS may be beneficial. Good basics in asthma management, such as effective management of comorbidities, ensuring that the patient learns and uses a good inhaler technique and gains control of symptoms with better adherence, would all reduce the need for the use of OCS.

Further studies are required to explore the direction of relationships between concerns about corticosteroids, experience of side effects and adherence, and to explore differences between patient and clinician perceptions of corticosteroids using clinician–patient dyads.

### Conclusions

This preliminary study identified a higher-than-expected frequency of side effects from ICS and OCS, and indicated that there may be disconnects between patient experiences of side effects and awareness of side effects among clinicians. Side effects were associated with strong concerns about corticosteroids, which, in turn, were associated with non-adherence. These findings have implications for the design of interventions to support patients, improve treatment experiences and enhance asthma control.

## Figures and Tables

**Table 1:** Demographics and scale summary scores for people taking ICS and OCS: comparison of those with complete and missing data

	*Prescribed ICS,* n*=2,213*^a^	*Complete data,* n*=1,524*	*Missing data,*^a^ n*=689*	P-*value*
Age (mean, s.d.)	54.2 (17.8)	52.7 (17.6)	58.1 (17.8)	0.000
Female (*n*,%)	1558 (73.1)	1080 (70.9)	478 (78.9)	0.000
MARS (mean, s.d.)	17.4 (3.2)	17.4 (3.2)	17.6 (3.2)	0.153
Side effects (mean, s.d.)	1.4 (1.4)	1.3 (1.42)	1.3 (1.5)	0.793
Concerns (mean, s.d.)	2.6 (0.7)	2.5 (0.7)	2.8 (0.7)	0.000
				
	*Prescribed OCS,* n*=314*^a^	*Complete data,* n*=233*	*Missing data,*^a^ n*=81*	P-*value*
Age (mean, s.d.)	50.1 (19.9)	48.5 (19.5)	56.1 (20.0)	0.006
Female (*n*,%)	244 (80.0)	186 (79.8)	58 (80.6)	0.893
MARS (mean, s.d.)	22.9 (2.9)	23.0 (2.6)	22.7 (2.6)	0.403
Side effects (mean, s.d.)	2.7 (1.7)	2.7 (1.7)	2.6 (1.8)	0.770
Concerns (mean, s.d.)	3.4 (0.7)	3.4 (0.6)	3.4 (0.7)	0.457

Abbreviations: ICS, inhaled corticosteroid; MARS, Medication Adherence Report Scale; OCS, oral corticosteroid.

a Summary statistics were calculated on data available, sample size within the overall sample and missing data columns therefore differs for each variable.

**Table 2:** Types of clinicians who responded to the online questionnaire

*Types of clinician*	*Total responded (*n* =534)*^a^	*Total included (*n* =244)*
GP	66 (12.4)	25 (10.2)
Practice nurse	297 (55.6)	164 (67.2)
Community nurse	42 (7.9)	11 (4.5)
Community pharmacist	24 (4.5)	8 (3.3)
Total primary care	429 (80.4)	208 (85.2)
Hospital doctor	9 (1.7)	4 (1.6)
Hospital pharmacist	6 (1.1)	1 (0.4)
Hospital nurse	32 (6.0)	6 (2.5)
Specialist nurse	58 (10.9)	25 (10.2)
Total secondary care	105 (19.7)	36 (14.8)

a 534/700 (76.3%) people who initiated the online questionnaire provided data on their profession.

## Notes

GlaxoSmithKline plc, MSD and Novartis Pharmaceuticals UK Ltd had no input into the study design, data collection, analysis or interpretation of the findings. RH has undertaken speaker engagements with honoraria with the following companies: Abbvie, Amgen, Biogen Idec, Gilead Sciences, GlaxoSmithKline, Janssen, Pfizer, Roche and Shire Pharmaceuticals. RH is funder and shareholder of a UCL-business spin out company (Spoonful of Sugar) providing consultancy on medication-related behaviours to healthcare policy makers, providers and industry. SW is an Associate editor of *npj Primary Care Respiratory Medicine*, but was not involved in the editorial review of, nor the decision to publish, this article. The other authors declare no conflicts of interest.
